# Entropy Generation Analysis and Natural Convection in a Nanofluid-Filled Square Cavity with a Concentric Solid Insert and Different Temperature Distributions

**DOI:** 10.3390/e20050336

**Published:** 2018-05-03

**Authors:** Ammar I. Alsabery, Muhamad Safwan Ishak, Ali J. Chamkha, Ishak Hashim

**Affiliations:** 1 Refrigeration & Air-conditioning Technical Engineering Department, College of Technical Engineering, The Islamic University, Najaf 54001, Iraq; 2 Center for Modelling & Data Science, Faculty of Science & Technology, Universiti Kebangsaan Malaysia, UKM Bangi 43600, Selangor, Malaysia; 3 Department of Engineering, College of Foundation and Diploma Studies, Universiti Tenaga Nasional, Kajang 43000, Selangor, Malaysia; 4 Department of Mechanical Engineering, Prince Sultan Endowment for Energy and the Environment, Prince Mohammad Bin Fahd University, Al-Khobar 31952, Saudi Arabia; 5 RAK Research and Innovation Center, American University of Ras Al Khaimah, P.O. Box 10021, Ras Al Khaimah 86416, UAE

**Keywords:** natural convection, entropy generation, finite difference method, nanofluid, inner solid insert, square cavity

## Abstract

The problem of entropy generation analysis and natural convection in a nanofluid square cavity with a concentric solid insert and different temperature distributions is studied numerically by the finite difference method. An isothermal heater is placed on the bottom wall while isothermal cold sources are distributed along the top and side walls of the square cavity. The remainder of these walls are kept adiabatic. Water-based nanofluids with Al${}_{2}$O${}_{3}$ nanoparticles are chosen for the investigation. The governing dimensionless parameters of this study are the nanoparticles volume fraction (0≤ϕ≤0.09), Rayleigh number $\left(10^{3}\leq Ra\leq 10^{6}\right)$, thermal conductivity ratio ($0.44\leq K_{r}\leq 23.8$) and length of the inner solid (0≤D≤0.7). Comparisons with previously experimental and numerical published works verify a very good agreement with the proposed numerical method. Numerical results are presented graphically in the form of streamlines, isotherms and local entropy generation as well as the local and average Nusselt numbers. The obtained results indicate that the thermal conductivity ratio and the inner solid size are excellent control parameters for an optimization of heat transfer and Bejan number within the fully heated and partially cooled square cavity.

## 1. Introduction

Fluid flow and heat transfer analysis in cavities have become popular areas of research related to heat storage applications. As a result, the increase in research activities regarding convection heat transfer is significant. When the fluid motion is not generated by any external source, the flow is characterized by natural convection flow. Such a mechanism is easily identified in various engineering applications such as electronic cooling, cooling of containment buildings, room ventilation, heat exchangers, storage tanks, double pane windows, solar collectors, and so on. Many researchers are interested in the use of nanofluids in the natural convection flow because of nanofluids’ excellent and adjustable properties including high thermal conductivity, stability, homogeneity and minimal clogging in flow passages due to small sizes and very large specific surface areas of the nano-sized particles. Khanafer et al. [1] have studied natural convection inside nanofluid-filled rectangular cavities. With the use of the finite-volume approach, the effect of metallic nanoparticles on the flow behaviour was studied, as well as the distribution of the heat transfer along with the thermal conductivity enhancement. It was found that the suspended nanoparticles strong enhancement was produced on the heat transfer rate for all values of the Grashof number. Similar findings were also found by Jou and Tzeng [2] who carried out a numerical study of natural convection heat transfers in nanofluids occupying a rectangular cavity using the finite-difference approach. Santra et al. [3] have studied the natural convection heat transfer of copper-water nanofluid consideration of Ostwald-de model based in a differentially heated square cavity. The finite volume approach was employed to study the enhancement of the heat transfer by using a copper particle with 100 nm size with a clear fluid. It was concluded that the heat transfer was reduced with an increase of the solid volume fraction for a particular value of the Rayleigh number. On the other hand, Alloui et al. [4] conducted a study on the natural convection in a shallow rectangular cavity filled with nanofluids. An excellent review on natural convective heat transfer in cavities with localized heating and filled with pure fluids or with nanofluids was reported by Öztop et al. [5]. To analyse the optimization of the fractal-like architecture of porous fibrous materials related to permeability, diffusivity, and thermal conductivity, Xiao et al. [6] used an established theoretical model called fractal analytical. The group discovered that the ratio of the dimensionless total effective thermal conductivity over dimensionless effective diffusivity was an increasing function of tortuosity fractal dimension. Recently, Alsabery et al. [7] investigated the effect of spatial side-wall temperature variation on transient natural convection of a nanofluid in a trapezoidal cavity by using the finite difference method numerically. The results revealed that the rate of the heat transfer was an increasing function of the nanoparticle volume fraction. Using the Fractal-Monte Carlo technique, Xiao et al. [8] conducted a research study on the relative permeability of nanofibers. They investigated different parameters, for example, saturation, porosity, area fractal dimension for pores, the fractal dimension of tortuous capillaries, capillary pressure and microstructural of porous nanofibers. Based on an experimental verification, Long and Xu [9] derived a perforation-erosion model and studied the effect of this model on dimensions of the fracture, distribution of the fluid and the drop of the pressure. Very recently, Xiao et al. [10] analytically studied the problem of water flow through unsaturated porous rocks using a derived model with the calculation of the capillary pressure and water relative permeability. Based on studies mentioned above and to the authors’ best knowledge, no work has been reported on the problem of natural convection fluid flow and heat transfer in a nanofluid-filled square cavity with different temperature distributions.

In the last two decades, many researchers have focused their attention on studying the convective heat transfer within cavities with one or more obstacles inside. House et al. [11] have considered the effect of a centered heat-conducting square body on natural convection heat transfer in a square cavity. In this study, two vertical walls were maintained at two different constant temperatures and the horizontal walls were adiabatic. Heat transfer was found to have decreased with the increase of the solid body size. Ha et al. [12] have numerically investigated the effect of unsteady natural convective heat transfer processes in similar vertical cavities with a centered heat-conducting body. Mahmoodi and Sebdani [13] also looked into the problem of natural convective flow and heat transfer of Cu-water nanofluid inside a square cavity having an adiabatic square bodies at its center. The results obtained showed that for all values of the Rayleigh number investigated with the exception of $Ra=10^{4}$, the average Nusselt number increased with an increase in the volume fraction of the nanoparticles. At $Ra=10^{4}$, the average Nusselt number was a decreasing function of the nanoparticles volume fraction. Moreover, the rate of heat transfer decreased when the size of the adiabatic square body increased at low Rayleigh numbers, whereas an increase in the heat transfer rate was observed at high Rayleigh numbers. Alsabery et al. [14] have numerically studied the problem of transient natural convective heat transfer in a nanofluid-saturated porous square cavity with a concentric solid insert and sinusoidal boundary condition. The right wall was heated sinusoidally, while the left vertical wall of the cavity was maintained at a constant temperature and a solid square was inserted at the center of the cavity. The results showed that the overall heat transfer was significantly increased with relatively non-uniform heating. Mehmood et al. [15] on the other hand, studied the mixed convection in alumina-water nanofluid lid-driven square cavity with an isothermally heated square blockage inside in the presence of a magnetic field. Using the Galerkin finite element method, they considered the effect of Reynolds number, Richardson number, nanoparticles volume fraction, Hartmann number and Eckert number. They found an enhancement on the average Nusselt number and kinetic energy was obtained when nanoparticles volume fraction was increased. Nevertheless, the study of natural convection heat transfer in a cavity with an inner solid insert and different temperature distributions has not yet been reported.

Many researchers have applied the heat transfer and entropy generation in various cavities using different methods. Kaluri and Basak [16] have studied the entropy generation numerically in porous square cavities with distributed heated sources during laminar natural convection in fluid saturated porous medium. They considered the effect of the permeability of the porous medium, the distributed heating technique and Prandtl number by using the Galerkin finite element method. Using the finite volume method, Mahapatra et al. [17] have numerically considered the study of heat transfer enhancement and entropy generation in a square cavity. This was done with the presence of an adiabatic block. Their results concluded that the heat transfer rate showed an enhancement with the increasing of the block size for low Rayleigh numbers. Lam and Prakash [18] studied the problem of natural convection and entropy generation in a porous cavity with heat sources using the finite element method. They investigated the effect of heat sources, Darcy number, porosity of the media and Rayleigh number. They then discovered that the rate of the heat transfer was an increasing function of Rayleigh number. Sheremet et al. [19] numerically took into consideration the entropy generation analysis on natural convection of nanofluid inside a square cavity having hot solid block with the use of Tiwari and Das’ model. Kefayati [20] have analyzed the heat transfer and entropy generation of laminar convection of non-Newtonian nanofluids in a porous cavity by the finite difference lattice Boltzmann method. Kolsi et al. [21] have considered the natural convection and entropy generation in an open cavity nanofluid filled with an adiabatic diamond shaped obstacle using the finite volume technique. Ismael et al. [22] have investigated the entropy generation due to conjugate natural convection-conduction heat transfer in a porous cavity heated by a triangular solid wall and saturated with a CuO–water nanofluid. Their results showed that both the average Nusselt number and the entropy generation were increasing functions of the thermal conductivity ratio, while there were maxima at some critical values of the ratio of the length of the inner solid compared to that of the outer hollow. Bouchoucha et al. [23] have investigated the entropy generation due to natural convection in a nanofluid-filled square cavity with a thick bottom wall. They found that the average Nusselt number decreased with increasing values of the thickness of the bottom wall. Meanwhile, the local heat transfer decreases with increasing wall thickness. Ghasemi and Siavashi [24] have reported a numerical study on natural convection in a porous cavity filled with Cu-water nanofluid using the Lattice Boltzmann method (LBM). Ashorynejad and Hoseinpour [25] have investigated the entropy generation analysis in the porous square cavity filled with nanofluid in natural convection using LBM considering a wide range of porosity and constant Darcy numbers with various volume fractions of nanoparticles.

Based on the studies mentioned above and to the authors’ best knowledge, the problem of entropy generation analysis and natural convection in a nanofluid-filled square cavity with a concentric solid insert and different temperature distributions is yet to be investigated. Hence, this study aims to investigate the entropy generation analysis and natural convection in nanofluid-filled square cavity with a concentric solid insert and different temperature distributions. A square cavity with an isothermal hot bottom wall and partly cold sidewalls and partly cold top wall are important problems in thermal processing applications, for example, in molten metals infiltration, drying and transport of gases, enhanced oil recovery by hot-water flooding and combustion of heavy oils [16,26]. Therefore, the authors believe that the present work is a valuable contribution in improving the thermal performance and the heat transfer enhancement in some engineering instruments.

## 2. Mathematical Formulation

**Figure 1:**
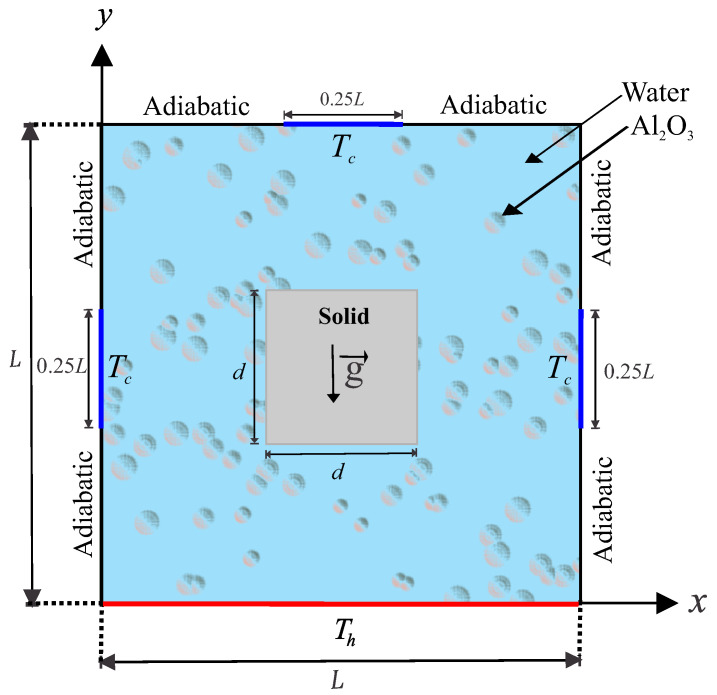
Physical model of convection in a square cavity together with the coordinate system.

The steady two-dimensional natural convection problem in a square cavity with length *L* and with the cavity center inserted by a solid square with side *d*, as illustrated in Figure 1.

The Rayleigh number range chosen in the study keeps the nanofluid flow laminar and two-dimensional. Isothermal cold sources are distributed along the top wall and side walls of the square cavity at a constant cold temperature $T_{c}$, which are showed by thick blue lines with length of 0.25L. While the remainder of these walls are kept adiabatic. The bottom wall of the cavity is maintained at a constant hot temperature $T_{h}$. The boundaries of the annulus are assumed to be impermeable; the fluid within the cavity is a water-based nanofluid having Al${}_{2}$O${}_{3}$ nanoparticles. The Boussinesq approximation is applicable. By considering these assumptions, the continuity, momentum and energy equations for the laminar and steady state natural convection can be written as follows [7,27]:

(1) $$\begin{array}{c} \frac{\partial u}{\partial x}+\frac{\partial v}{\partial y}=0, \end{array}$$

(2) $$\begin{array}{c} u\frac{\partial u}{\partial x}+v\frac{\partial u}{\partial y}=-\frac{1}{\rho_{nf}}\frac{\partial p}{\partial x}+\frac{\mu_{nf}}{\rho_{nf}}\left(\frac{\partial^{2}u}{\partial x^{2}}+\frac{\partial^{2}u}{\partial y^{2}}\right), \end{array}$$

(3) $$\begin{array}{c} u\frac{\partial v}{\partial x}+v\frac{\partial v}{\partial y}=-\frac{1}{\rho_{nf}}\frac{\partial p}{\partial y}+\frac{\mu_{nf}}{\rho_{nf}}\left(\frac{\partial^{2}v}{\partial x^{2}}+\frac{\partial^{2}v}{\partial y^{2}}\right)+\beta_{nf}g\left(T_{h}-T_{c}\right), \end{array}$$

(4) $$\begin{array}{c} u\frac{\partial T_{nf}}{\partial x}+v\frac{\partial T_{nf}}{\partial y}=\alpha_{nf}\left(\frac{\partial^{2}T_{nf}}{\partial x^{2}}+\frac{\partial^{2}T_{nf}}{\partial y^{2}}\right). \end{array}$$

The energy equation of the inner solid wall is

(5) $$\frac{\partial^{2}T_{w}}{\partial x^{2}}+\frac{\partial^{2}T_{w}}{\partial y^{2}}=0,$$

where *x* and *y* are the Cartesian coordinates measured in the horizontal and vertical directions respectively, *g* is the acceleration due to gravity, $\alpha_{nf}$ is the effective thermal diffusivity of the nanofluids and $\rho_{nf}$ is the effective density of the nanofluids, which are defined as [28]

(6) $$\begin{array}{c} \alpha_{nf}=\frac{k_{nf}}{{\left(\rho C_{p}\right)}_{nf}},\\ \rho_{nf}=\left(1-\phi \right)\rho_{bf}+\phi \rho_{sp}, \end{array}$$

where ϕ is the solid volume fraction of nanoparticles and the heat capacitance of the nanofluids ${\left(\rho C_{p}\right)}_{nf}$ is given as [28]

(7) $${\left(\rho C_{p}\right)}_{nf}=\left(1-\phi \right){\left(\rho C_{p}\right)}_{bf}+\phi {\left(\rho C_{p}\right)}_{sp}.$$

The thermal expansion coefficient of the nanofluids can be determined by [28]:

(8) $$\begin{array}{c} \beta_{nf}=\left(1-\phi \right){\left(\beta \right)}_{bf}+\phi \beta_{sp}, \end{array}$$

(9) $$\begin{array}{c} {\left(\rho \beta \right)}_{nf}=\left(1-\phi \right){\left(\rho \beta \right)}_{bf}+\phi {\left(\rho \beta \right)}_{sp}. \end{array}$$

The dynamic viscosity ratio of Al${}_{2}$O${}_{3}$–water nanofluids for 36 nm particle-size in the ambient condition was derived by Nguyen et al. [29] as follows:

(10) $$\frac{\mu_{nf}}{\mu_{bf}}=1+0.025\phi +0.015\phi^{2},$$

where the experimental work of Nguyen et al. [29] is valid for temperatures ranging from 20 to 75 ${}^{\circ }$C, for water–Al${}_{2}$O${}_{3}$ mixtures with two different particle diameters, 36 nm and 47 nm, as well as for water–CuO nanofluid with 29 nm particle size. While the range of the particle volume fraction is taken from 0 to 0.14.

The temperature dependence of water viscosity $\mu_{bf}$ is expressed by Chon et al. [30] as

(11) $$\mu_{bf}=2.414\times 10^{-5}10^{247.8/(T-140)}.$$

The thermal conductivity ratio of Al${}_{2}$O${}_{3}$–water nanofluids calculated by the Chon model [30] is

(12) knfkbf=1+64.7ϕ0.7640dbfdsp0.7476PrT0.9955Re1.2321.

This model is valid for temperatures ranging from 21 to 71 ${}^{\circ }$C, for water–Al${}_{2}$O${}_{3}$ mixtures for different particle diameters from 11 nm to 150 nm, and the range of the particle volume fraction from 0 to 0.14. Here ${\mathrm{Pr}}_{T}$ and Re are defined as

(13) $${\mathrm{Pr}}_{T}=\frac{\mu_{bf}}{\rho_{bf}\alpha_{bf}}.$$

(14) Re=ρbfkbT3πμbf2lbf,

where $k_{b}=1.380648\times 10^{-23}$ (J/K) is the Boltzmann constant, $l_{bf}=0.17$ nm is the mean path of the used fluid (water) for the entire tested temperature range (from 21 to 71 ${}^{\circ }$C) [30], and $d_{bf}$ is the molecular diameter of water given by Corcione [31]

(15) $$d_{bf}=\frac{6M}{N\pi \rho_{bf}}.$$

Here *M* is the molecular weight of the base fluid, *N* is the Avogadro number and $\rho_{bf}$ is the density of the base fluid at standard temperature (310 K). Accordingly and basing on water as a base fluid, the value of $d_{bf}$ is [31]:

(16) $$d_{bf}={\left(\frac{6\times 0.01801528}{6.022\times 10^{23}\times \pi \times 998.26}\right)}^{1/3}=3.85\times 10^{-10}m.$$

Now define the stream function ψ and the vorticity ω in the usual way

(17) $$\begin{array}{c} u=\frac{\partial \psi }{\partial y},\;\;v=-\frac{\partial \psi }{\partial x}, \end{array}$$

(18) $$\begin{array}{c} \omega =\frac{\partial v}{\partial x}-\frac{\partial u}{\partial y}, \end{array}$$

and introduce the following non-dimensional variables:

(19) $$\begin{array}{cc} & X=\frac{x}{L},\;\;Y=\frac{y}{L},\;\;\Omega =\frac{\omega L^{2}}{\alpha_{bf}},\;\;\Psi =\frac{\psi }{\alpha_{bf}},\;\;\theta_{nf}=\frac{T_{nf}-T_{c}}{T_{h}-T_{c}},\;\;\theta_{w}=\frac{T_{w}-T_{c}}{T_{h}-T_{c}}. \end{array}$$

Substituting the non-dimensional variables (19) into the governing Equations (1)–(5) we obtain the following dimensionless equations:

(20) $$\begin{array}{c} \frac{\partial^{2}\Psi }{\partial X^{2}}+\frac{\partial^{2}\Psi }{\partial Y^{2}}=-\Omega , \end{array}$$

(21) $$\begin{array}{c} \frac{\partial \Psi }{\partial Y}\frac{\partial \Omega }{\partial X}-\frac{\partial \Psi }{\partial X}\frac{\partial \Omega }{\partial Y}=\frac{\mu_{nf}}{\mu_{bf}}\left[\frac{\mathrm{Pr}}{\left(1-\phi \right)+\phi \frac{\rho_{sp}}{\rho_{bf}}}\right]\left(\frac{\partial^{2}\Omega }{\partial X^{2}}+\frac{\partial^{2}\Omega }{\partial Y^{2}}\right)+\frac{\beta_{nf}}{\beta_{bf}}Ra\mathrm{Pr}\left(\frac{\partial \theta }{\partial X}\right), \end{array}$$

(22) $$\begin{array}{c} \frac{\partial \Psi }{\partial Y}\frac{\partial \theta_{nf}}{\partial X}-\frac{\partial \Psi }{\partial X}\frac{\partial \theta_{nf}}{\partial Y}=\frac{{\left(\rho C_{p}\right)}_{bf}}{{\left(\rho C_{p}\right)}_{nf}}\frac{k_{nf}}{k_{bf}}\left(\frac{\partial^{2}\theta_{nf}}{\partial X^{2}}+\frac{\partial^{2}\theta_{nf}}{\partial Y^{2}}\right), \end{array}$$

(23) $$\begin{array}{c} \frac{\partial^{2}\theta_{w}}{\partial X^{2}}+\frac{\partial^{2}\theta_{w}}{\partial Y^{2}}=0, \end{array}$$

where $Ra=g\rho_{bf}\beta_{bf}\left(T_{h}-T_{c}\right)L^{3}/(\mu_{bf}\alpha_{bf}$) is the Rayleigh number for the base fluid and $\mathrm{Pr}=\nu_{bf}/\alpha_{bf}$ is the Prandtl number for the base fluid. The dimensionless boundary conditions of Equations (20) and (23) are:

(24) $$\begin{array}{ccc} \Psi & = & 0,\;\;\mathrm{on}\;\mathrm{all}\;\mathrm{solid}\;\mathrm{boundaries}, \end{array}$$

(25) $$\begin{array}{ccc} \theta_{nf}\left(X,0\right) & = & 1,\;\;\left(\mathrm{for}\;\mathrm{the}\;\mathrm{hot}\;\mathrm{bottom}\;\mathrm{wall}\right), \end{array}$$

(26) $$\begin{array}{ccc} \theta_{nf}\left(X,Y\right) & = & 0,\;\;\left(\mathrm{for}\;\mathrm{the}\;\mathrm{cold}\;\mathrm{regimes}\;\mathrm{in}\;\mathrm{the}\;\mathrm{side}\;\mathrm{and}\;\mathrm{top}\;\mathrm{walls}\right) \end{array}$$

(27) $$\begin{array}{ccc} \frac{\partial \theta (X,Y)}{\partial Y} & = & 0,\;\;\left(\mathrm{for}\;\mathrm{the}\;\mathrm{adiabatic}\;\mathrm{part}\;\mathrm{of}\;\mathrm{the}\;\mathrm{remainder}\;\mathrm{walls}\right) \end{array}$$

(28) $$\begin{array}{ccc} \theta_{nf} & = & \theta_{w},\;\;\mathrm{at}\;\mathrm{the}\;\mathrm{outer}\;\mathrm{solid}\;\mathrm{square}\;\mathrm{surface}, \end{array}$$

(29) $$\begin{array}{ccc} \frac{\partial \theta_{nf}}{\partial X} & = & K_{r}\frac{\partial \theta_{w}}{\partial X},\;\;\left|X\right|\leq D, \end{array}$$

(30) $$\begin{array}{ccc} \frac{\partial \theta_{nf}}{\partial Y} & = & K_{r}\frac{\partial \theta_{w}}{\partial Y},\;\;\left|Y\right|\leq D, \end{array}$$

where $K_{r}=k_{w}/k_{nf}$ is the thermal conductivity ratio and D=d/L is the aspect ratio of inner square cylinder width to outer square cylinder width.

The local Nusselt number evaluated at the bottom wall is defined by

(31) $$Nu_{nf}=-\frac{k_{nf}}{k_{bf}}{\left(\frac{\partial \theta_{nf}}{\partial Y}\right)}_{Y=0}.$$

Finally, the average Nusselt number evaluated at the bottom wall which is given by:

(32) $$\begin{array}{c} {\bar{Nu}}_{nf}=\int_{0}^{1}Nu_{nf}\;dX. \end{array}$$

The entropy generation relation is given by

(33) $$\begin{array}{c} S=\frac{k_{nf}}{T_{0}^{2}}\left[{\left(\frac{\partial T}{\partial x}\right)}^{2}+{\left(\frac{\partial T}{\partial y}\right)}^{2}\right]+\frac{\mu_{nf}}{T_{0}}\left[2{\left(\frac{\partial u}{\partial x}\right)}^{2}+2{\left(\frac{\partial v}{\partial y}\right)}^{2}+{\left(\frac{\partial u}{\partial x}+\frac{\partial v}{\partial x}\right)}^{2}\right]. \end{array}$$

In dimensionless form, the local entropy generation can be expressed as:

(34) $$\begin{array}{c} S_{\mathrm{GEN}}=\frac{k_{nf}}{k_{bf}}\left[{\left(\frac{\partial \theta }{\partial X}\right)}^{2}+{\left(\frac{\partial \theta }{\partial Y}\right)}^{2}\right]+\frac{\mu_{nf}}{\mu_{bf}}N_{\mu }\left[4{\left(\frac{\partial^{2}\Psi }{\partial X\partial Y}\right)}^{2}+{\left(\frac{\partial^{2}\Psi }{\partial Y^{2}}-\frac{\partial^{2}\Psi }{\partial X^{2}}\right)}^{2}\right], \end{array}$$

where, $N_{\mu }=\frac{\mu_{bf}T_{0}}{k_{bf}}{\left(\frac{\alpha_{bf}}{L(\Delta T)}\right)}^{2}$ is the irreversibility distribution ratio and $S_{\mathrm{GEN}}=S_{\mathrm{gen}}\frac{T_{0}^{2}L^{2}}{k_{bf}{\left(\Delta T\right)}^{2}}$. The terms of Equation (34) can be rewritten in the following form:

(35) $$\begin{array}{c} S_{\mathrm{GEN}}=S_{\theta }+S_{\Psi }, \end{array}$$

where $S_{\theta }$ and $S_{\Psi }$ are the entropy generation due to heat transfer irreversibility (HTI) and nanofluid friction irreversibility (NFI), respectively,

(36) $$\begin{array}{cc} & S_{\theta }=\frac{k_{nf}}{k_{bf}}\left[{\left(\frac{\partial \theta }{\partial X}\right)}^{2}+{\left(\frac{\partial \theta }{\partial Y}\right)}^{2}\right], \end{array}$$

(37) $$\begin{array}{c} S_{\Psi }=\frac{\mu_{nf}}{\mu_{bf}}N_{\mu }\left[4{\left(\frac{\partial^{2}\Psi }{\partial X\partial Y}\right)}^{2}+{\left(\frac{\partial^{2}\Psi }{\partial Y^{2}}-\frac{\partial^{2}\Psi }{\partial X^{2}}\right)}^{2}\right]. \end{array}$$

By integrating Equation (35) over the domain, the global entropy generation (GEG) for the present two-dimensional study is obtained

(38) $$\begin{array}{c} \mathrm{GEG}=\int S_{\mathrm{GEN}}\;dXdY=\int S_{\theta }\;dXdY+\int S_{\Psi }\;dXdY. \end{array}$$

We mention now the Bejan number to display dominance between heat transfer or nanofluid friction irreversibility:

(39) $$\begin{array}{c} Be=\frac{\int S_{\theta }\;dXdY}{\int S_{\mathrm{GEN}}\;dXdY}. \end{array}$$

When Be>0.5, the heat transfer irreversibility (HTI) is dominant, while when Be<0.5, the nanofluid friction irreversibility (NFI) is dominant.

## 3. Numerical Method and Validation

The governing dimensionless Equations (20)–(23) subject to the boundary conditions (24)–() are solved numerically by the finite difference method (FDM). In the present paper, several grid testings are performed: 10×10, 20×20, 40×40, 60×60, 80×80, 100×100, 120×120, 140×140 and 160×160. Table 1 shows the calculated strength of the flow circulation ($\Psi_{\min}$) and average Nusselt number (${\bar{Nu}}_{nf}$) at different grid sizes for $Ra=10^{5}$, ϕ=0.04, $K_{r}=1$ and D=0.25. The results show insignificant differences for the 140×140 grids and above. Therefore, for all computations in this paper for similar problems to this subsection, the 140×140 uniform grid is employed.

**Figure 2:**
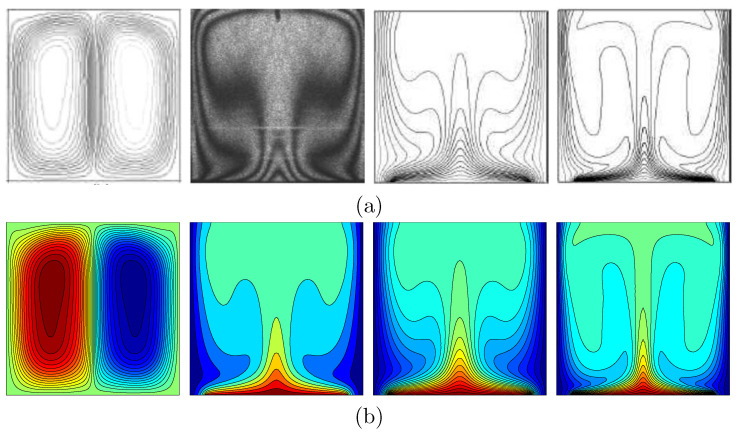
streamlines at $Ra=10^{6}$, isotherms at $Ra=1.836\times 10^{5}$, isotherms at $Ra=10^{5}$ and isotherms at $Ra=10^{6}$ (**a**) numerical and experimental results of Calcagni et al. [32] and (**b**) numerical results of the present study for ϕ=0 and D=0.

**Figure 3:**
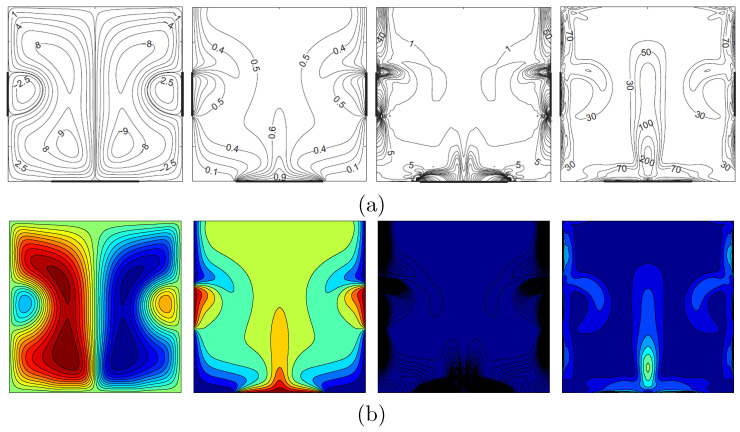
Streamlines, isotherms, local entropy generation due to heat transfer and due to fluid friction (**a**) Kaluri and Basak [16] and (**b**) present study for $Ra=10^{6}$, $Da=10^{-3}$, ϕ=0 and D=0.

For the validation of data, the results are compared with previously published numerical and experimental results obtained by Calcagni et al. [32] for the case of natural convection heat transfer in a square cavity partially heated from below, as shown in Figure 2. A second validation presented a comparison between the current results and previous published numerical results obtained by Kaluri and Basak [16] for the case of entropy generation analysis and natural convection in a discretely heated porous square cavity, as shown in Figure 3. These results provide confidence to the accuracy of the present numerical method.

## 4. Results and Discussion

In this section, we present numerical results for the streamlines, isotherms and isentropic lines (the local dimensionless entropy generation) with various values of the nanoparticles volume fraction (0≤ϕ≤0.09), the Rayleigh number $\left(10^{3}\leq Ra\leq 10^{6}\right)$, thermal conductivity ratio ($K_{r}=0.44$, 1, 2.40, 9.90 and 23.8) (epoxy-water: 0.44, brickwork-water: 1, glass-water: 2.40, epoxy-air: 9.90, stainless steel-water: 23.8) and the length of the inner solid (0≤D≤0.6). The values of the average Nusselt number, global entropy generation GEG, overall Bejan number Be are calculated for various values of Ra, ϕ and *D*. The thermophysical properties of the base fluid (water) and solid Al${}_{2}$O${}_{3}$ phases are tabulated in Table 2.

**Figure 4:**
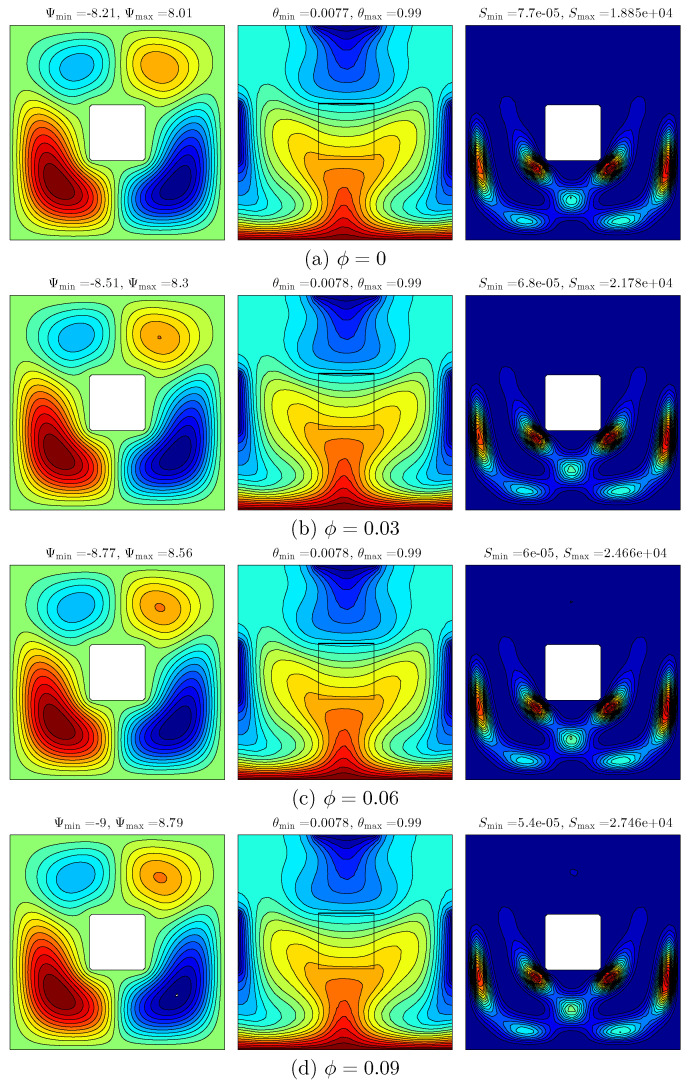
Variations of the streamlines (**left**); isotherms (**middle**); and isentropic (**right**) evolution by the solid volume fraction (ϕ) for $Ra=10^{5}$, $K_{r}=1$ and D=0.25. (**a**) ϕ=0; (**b**) ϕ=0.03; (**c**) ϕ=0.06; (**d**) ϕ=0.09.

Figure 4 presents the effect of various volume fraction of nanoparticles on the streamlines (left), isotherms (middle), and isentropic (right) evolution for $Ra=10^{5}$, $K_{r}=1$ and D=0.25. Heating the bottom wall of the cavity by a constant hot temperature and then distributing the isothermal cold sources along the top wall and side walls of the square cavity clearly have an effect on the temperature distribution and the flow behaviour. The flow inside the cavity appears with streamlined rotating cells: the two cells in the clockwise direction at the top left and bottom right of the cavity while another two cells in the anticlockwise direction are located at the top right and bottom left of the cavity. It can be seen that the high intensity located at the bottom of the cavity was affected by the constant exposure to high temperature on the bottom of the wall. Heating the bottom wall of the cavity causes the appearance of the curved lines in the isotherm patterns with high intensity in nanofluid. As the volume fraction of nanoparticles increases, the strength of the streamlines increases as well due to the effect of the viscosity forces and inertial forces. With the increase in volume fraction, the thermal conductivity of the fluid medium increases, resulting in heat transfer enhancement which then increases the strength of the flow. As a result, a new cell appears with an increase in size of the cell simultaneously with the increase in the nanoparticles’ volume fraction. Meanwhile, it is apparent that there is no discernible change in the isotherm patterns. The intensity of the isentropic patterns tends to increase with the increment of nanoparticles volume fraction resulting in the high thermal conductivity of nanoparticles.

**Figure 5:**
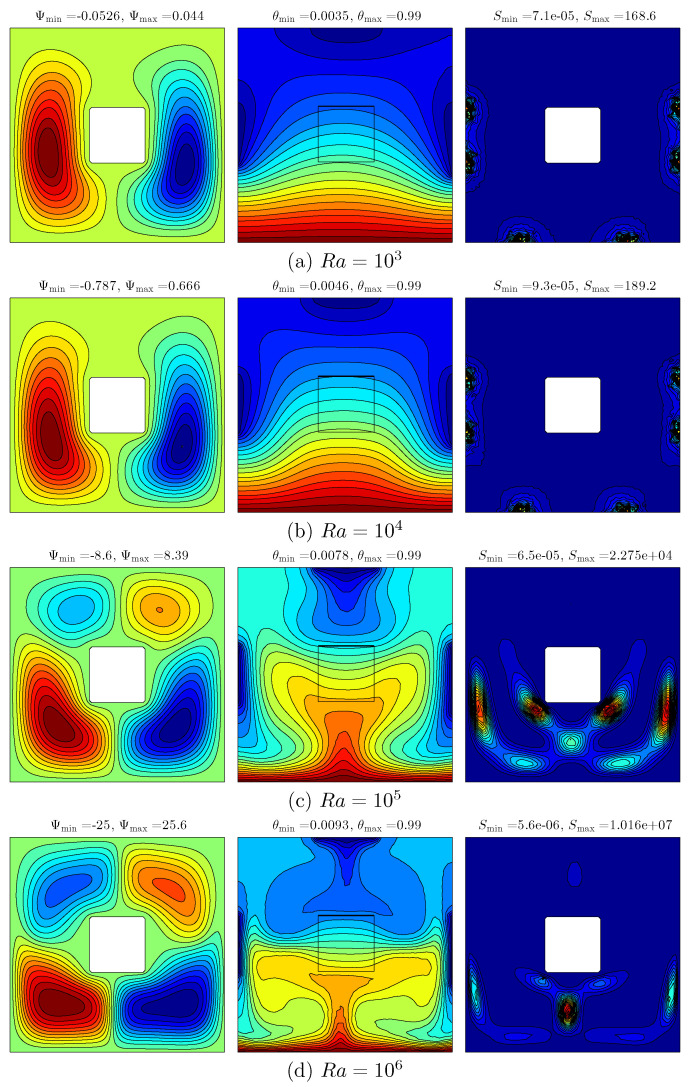
Variations of the streamlines (**left**); isotherms (**middle**); and isentropic (**right**) evolution by Rayleigh number (Ra) for ϕ=0.04, $K_{r}=1$ and D=0.25. (**a**) $Ra=10^{3}$; (**b**) $Ra=10^{4}$; (**c**) $Ra=10^{5}$; (**d**) $Ra=10^{6}$.

Figure 5 shows the result of various Rayleigh number on the streamlines (left), isotherms (middle) and isentropic (right) for ϕ=0.04, $K_{r}=1$ and D=0.25. At the low Ra value ($10^{3}$), the flow inside the cavity appears with two streamlined rotating cells, with an anticlockwise fluid heat flow on the left and solid square with a clockwise fluid heat flow on the right. This is due to the effect of vertical walls with the variation of the temperature distributions. The isotherm patterns appear to have taken curved lines at the cold sources and high intensity of isotherms at the bottom of the wall, showing the result of the hot source. The isentropic patterns appear with a spot shape at the right as well as the left edges of the cavity walls. As the Rayleigh number increases, the intensity of the streamlines along with the amount and size of the cells increase caused by the strong buoyancy forces compared to the viscous forces. The strength of the flow circulation increases with the Rayleigh numbers being affected by the increment of buoyancy force and convection intensity. At a higher Rayleigh number value, the flow behavior and temperature distribution are significantly affected, as shown in Figure 5d. It can be seen that the intensity of the isotherm patterns of the nanofluid increases and occupies more space within the cavity, and the nanofluid isotherm patterns appear at both the hot and cold sources. The intensity of the isotherm patterns within the inner solid also increased with the increase in the Rayleigh number. Even though the intensity of the isentropic patterns of the nanofluid increases until the Rayleigh numbers are at $10^{5}$, the intensity declines after the Rayleigh number is at $10^{6}$.

**Figure 6:**
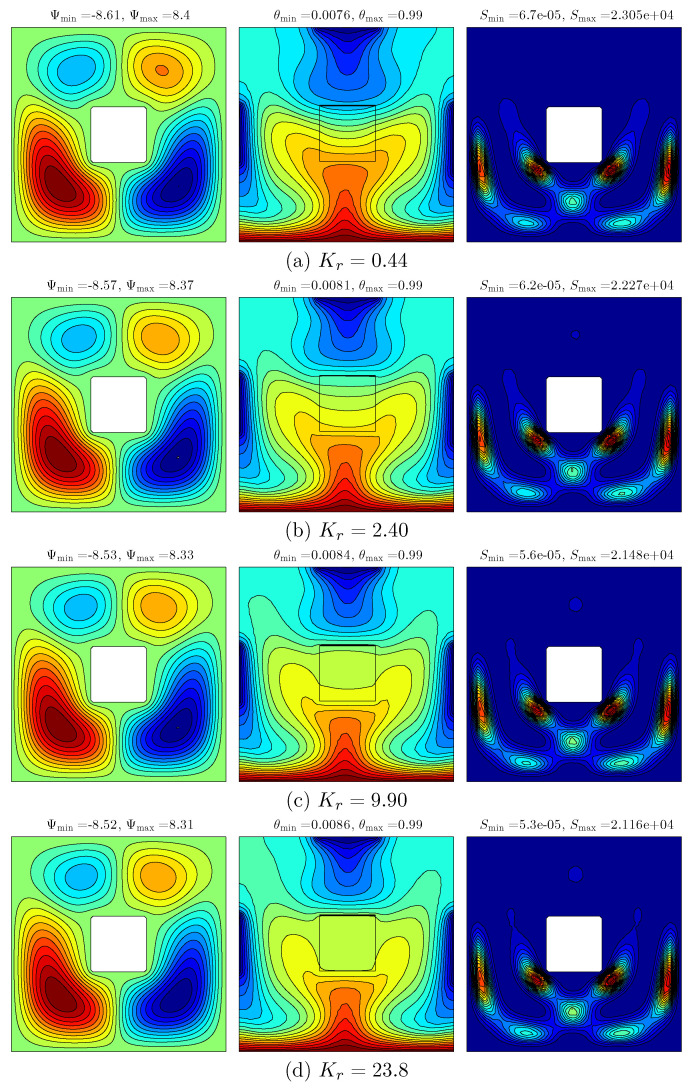
Variations of the streamlines (**left**); isotherms (**middle**); and isentropic (**right**) evolution by thermal conductivity ratio ($K_{r}$) for $Ra=10^{5}$, ϕ=0.04 and D=0.25. (**a**) $K_{r}=0.44$; (**b**) $K_{r}=2.40$; (**c**) $K_{r}=9.90$; (**d**) $K_{r}=23.8$.

Figure 6 demonstrates the effect of various thermal conductivity ratios on the streamlines (left), isotherms (middle) and isentropic (right) for $Ra=10^{5}$, ϕ=0.04 and D=0.25. At the low thermal conductivity ratio, it appears that four rotating cells are evident with a high intensity on the bottom wall of the cavity. The isotherm patterns appear with curved lines at the vertical and bottom walls of the cavity, while the isentropic appears with curved lines and high intensity at the bottom of the cavity. This is caused by the varied temperature distributions at the vertical and horizontal walls. As the thermal conductivity ratio increases, the strength and intensity of the streamlines decreases, while we observed that the cell at the top right vertical wall disappeared due to the fact that the resistance of the solid inner cavity tends to reduce the thermal conductivity ratio. In addition, the intensity of the isotherm patterns had decreased due to the reduction of thermal resistance of the solid inner square, which transferred more heat within the cavity by the increase of thermal conductivity ratio. Meanwhile, the intensity of isentropic patterns along with the size of cells increases as thermal conductivity ratio increases.

**Figure 7:**
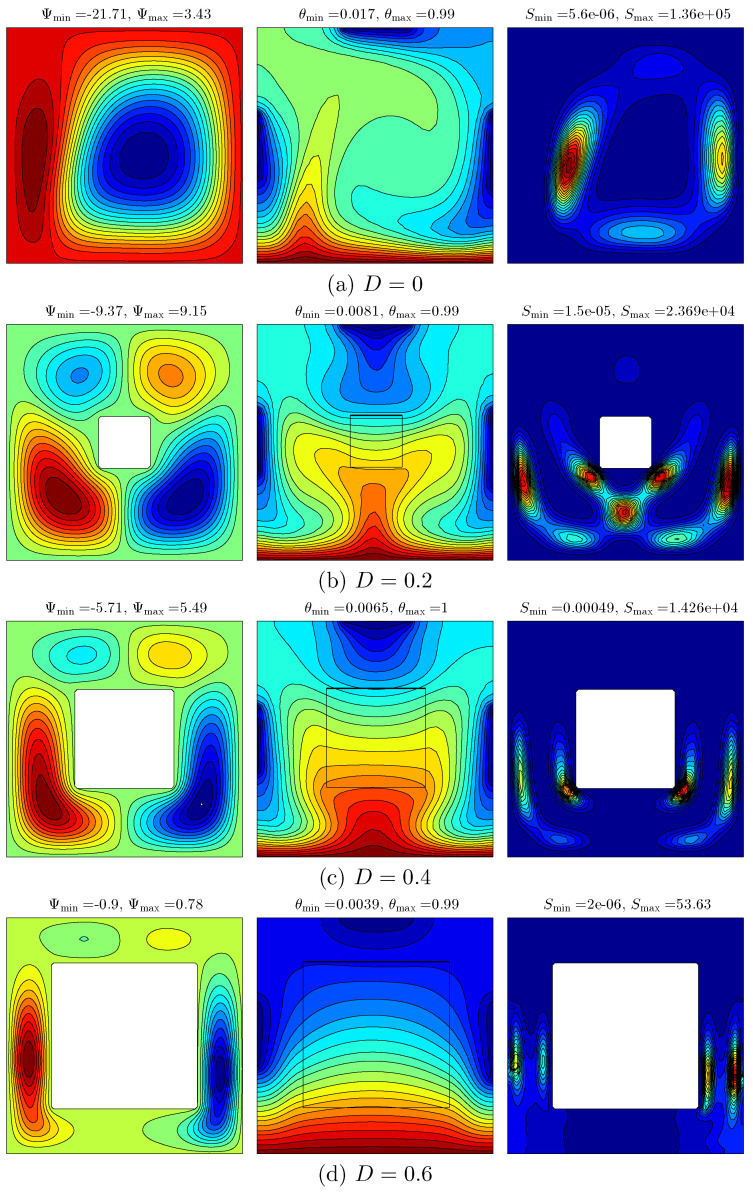
Variations of the streamlines (**left**); isotherms (**middle**); and isentropic (**right**) evolution by the length of the inner solid (*D*) for $Ra=10^{5}$, ϕ=0.04 and $K_{r}=1$. (**a**) D=0; (**b**) D=0.2; (**c**) D=0.4; (**d**) D=0.6.

Figure 7 depicts the effect of various lengths of the inner solid square on the streamlines (left), isotherms (middle) and isentropic (right) for $Ra=10^{5}$, ϕ=0.04 and $K_{r}=1$. Figure 7a shows the flow behavior and temperature distribution within the cavity in the absence of the inner solid square (D=0). It can be seen that the streamline within the cavity tends to appear with a primary cell located within the middle of the cavity, and with the secondary cell next to the left vertical side of the cavity. The minimum strength of the flow circulation decreases with the insertion of the inner solid square. On the contrary, the maximum strength of the flow circulation increases. This occurrence is affected by the viscosity and the inertial force in addition to the higher resistance of the inner solid. Consequently, increasing the length of the inner solid wall significantly affects the distribution of the flow and temperature profiles. The vertical expansion of the primary streamline allows another two cells to appear on the solid inner square. On the contrary, the secondary cell expands vertically with the increase of the inner solid length because of the thermal property of the solid insert. The intensity and number of isotherm patterns of the nanofluid are distinctly increased within the solid inner square. This is due to the difference between the thermal conductivity of the nanofluid and the thermal conductivity of the solid insert, resulting in more heat being received by the medium in which the thermal conductivity is relatively great, while the intensity of the isentropic lines reduces as the length of the inner cavity increases.

**Figure 8:**
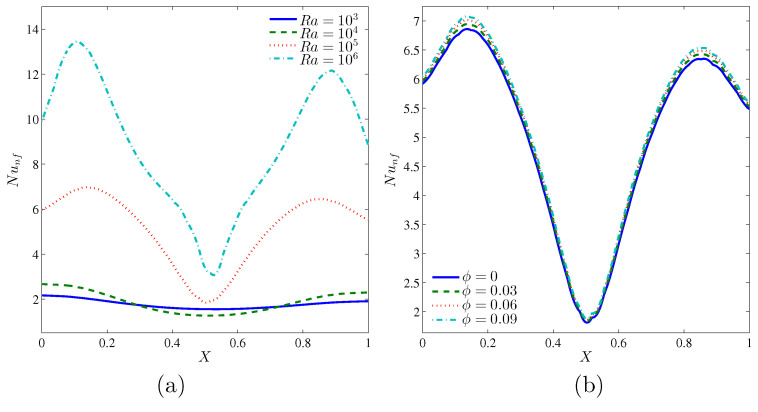
Variations of local Nusselt number interfaces with *X* for different (**a**) Ra and (**b**) ϕ at $K_{r}=1$ and D=0.25.

Figure 8 illustrates the effects of various values of the Rayleigh numbers and the volume fraction of nanoparticles on the local Nusselt number, and along the *X* coordinates for $K_{r}=1$ and D=0.25. Due to the hot source at the bottom wall and cold sources on the left, right and top walls of the cavity, the local Nusselt numbers tend to take a sinusoidal shape for all Rayleigh numbers and volume fraction of nanoparticles. Increasing the Rayleigh numbers enhances the local heat transfer as illustrated in Figure 8a. Meanwhile, the convective heat transfer increases with the augmentation of nanoparticles volume fraction, as illustrated in Figure 8b.

**Figure 9:**
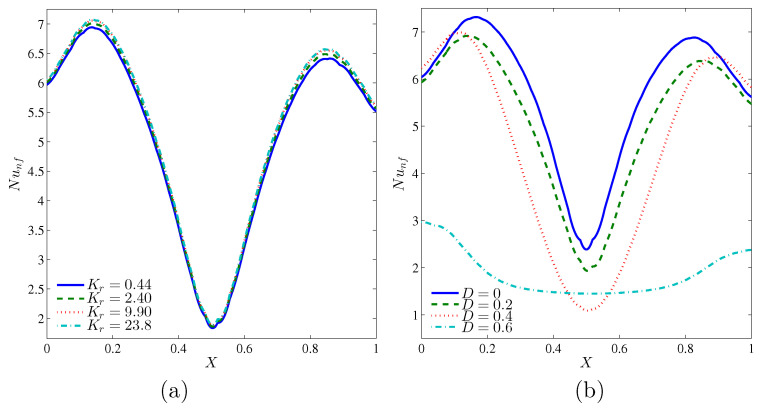
Variations of local Nusselt number interfaces with *X* for different (**a**) $K_{r}$ and (**b**) *D* at $Ra=10^{5}$ and ϕ=0.04.

Figure 9 illustrates the effects of various values of the thermal conductivity ratio and the length of inner cavity on the local Nusselt number, and along the *X* coordinates for $Ra=10^{5}$ and ϕ=0.04. Similar to the previous figure, the local Nusselt number has the sinusoidal shape for all thermal conductivity ratios and inner solid lengths, due to the varsity heating systems on the walls of the cavity. It can be seen that the maximum value of the local Nusselt number can be obtained at $K_{r}=2.40$, while the case shown in Figure 9b, with no inner solid in the cavity, gave the maximum value of the local Nusselt number due to the absence of the heat resistance by the solid inner square.

**Figure 10:**
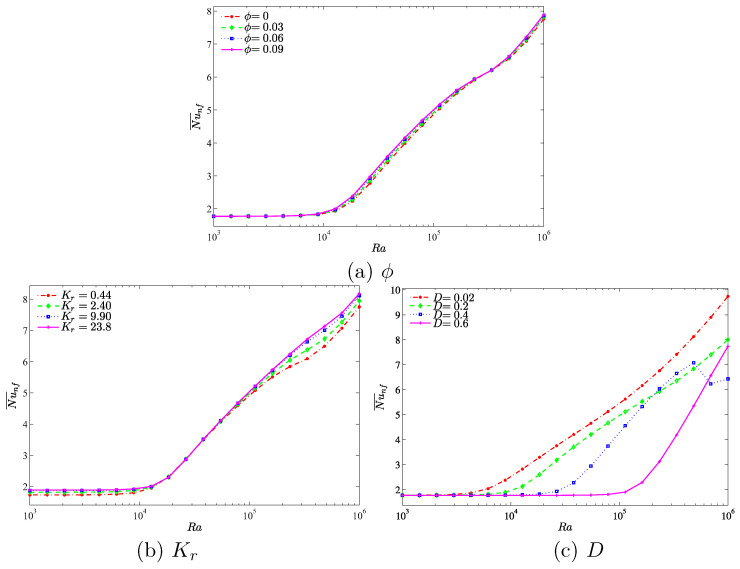
Variations of average Nusselt number with Ra for different (**a**) ϕ; (**b**) $K_{r}$ and (**c**) *D*.

A comprehensive picture about the overall heat transfer is illustrated by the average Nusselt number. Hence, Figure 10 indicates the average Nusselt number with different Rayleigh numbers on (a) nanoparticles volume fraction, (b) the thermal conductivity ratio and (c) the length of the inner cavity. We clearly observe that the convective heat transfer increases when the Rayleigh number is $10^{4}$ for all figures. This is due to the strong buoyancy forces compared to the viscous forces at higher Rayleigh numbers. In Figure 10a,b, the application of the higher nanoparticles volume fraction (a) and the higher thermal conductivity ratio (b) significantly increases the heat transfer rate, which results in a maximum average Nusselt number. Furthermore, the application of lower inner of cavity increases the heat transfer rate, which results in a maximum average Nusselt number. However, a diverse behavior appears on the heat transfer rate with the increase of Rayleigh number in [$4.83\times 10^{5},10^{6}$] for the case D=0.4. The rate of the heat transfer shows a decreasing function with an increment of Rayleigh number in [$4.83\times 10^{5},6.95\times 10^{5}$], while beyond that when the value of Ra is between [$6.95\times 10^{5},10^{6}$], the heat transfer rate tends to increase. This is due to the fact that the convection heat transfer is strongly enhanced by the inner solid. The gap between the cavity wall and the inner solid is decreased by a bigger inner solid insert. As a result, the fluid is clearly accelerated which increases the convection heat transfer for a relatively low Rayleigh number. However, as the Rayleigh number is increased further we observe a contrast in behavior. In the narrow gap between the wall and inner solid block, a relatively high Rayleigh number induces high velocity which in turn slows down the heat transfer process. The unexpected increase of the heat transfer at a very high Rayleigh number can be attributed to the onset of turbulent convection in the narrow space between the wall and the inner solid where the dynamics are spatio-temporally chaotic.

**Figure 11:**
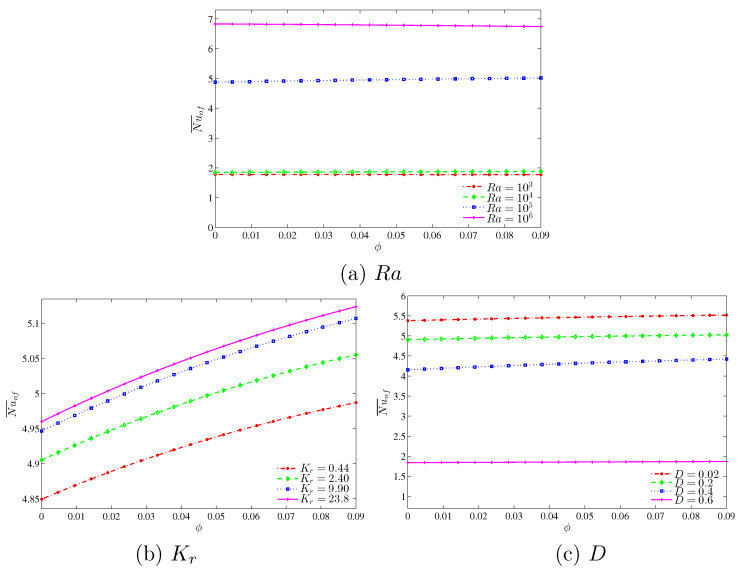
Variations of average Nusselt number with ϕ for different (**a**) Ra; (**b**) $K_{r}$ and (**c**) *D*.

In Figure 11, we observe an interesting result in relation to the influence of the Rayleigh numbers, thermal conductivity ratio and the length of the inner cavity on the average Nusselt number with different nanoparticles volume fractions. It is observed that the convective heat transfer is less affected by the increment of the nanoparticles volume fractions. However, the heat transfer rate increases with the increase in the Rayleigh numbers. Additionally, we observe a very strong enhancement on the rate of the heat transfer by increasing the Rayleigh number from $10^{4}$ to $10^{5}$ in comparison to other values of Rayleigh number. This is because the buoyancy forces increment compared to the viscous forces is very high in this range of Rayleigh number which clearly demonstrates significant enhancement on the heat transfer rate. Figure 11b illustrates the effect of various thermal conductivity ratios on the average Nusselt number with different nanoparticles volume fractions. As with the previous case, the overall heat transfer is less affected by the increase in nanoparticles volume fraction. The convective heat transfer is increased by the augmentation of nanoparticles volume fractions, which is attributed to a higher thermal conductivity of the nanoparticles. In addition, it is clearly observed that the rate of the heat transfer increases simultaneously with an increase in the thermal conductivity ratio due to the reduction of the thermal resistance which allows the transfer of more heat within the cavity. Figure 11c depicts the effect of various lengths of the inner solid square on the average Nusselt number with different nanoparticles volume fractions. The convective heat transfer is shown to increase with an increase in nanoparticles volume fraction due to the higher thermal conductivity. We observed that the heat transfer is less affected by the increment in the nanoparticles volume fraction. In addition to that, the overall heat transfer decreases with an increase in the length of the inner cavity due to higher heat resistance of the solid inner square, which then decreases the temperature gradient and hence there is a smaller heating effect.

**Figure 12:**
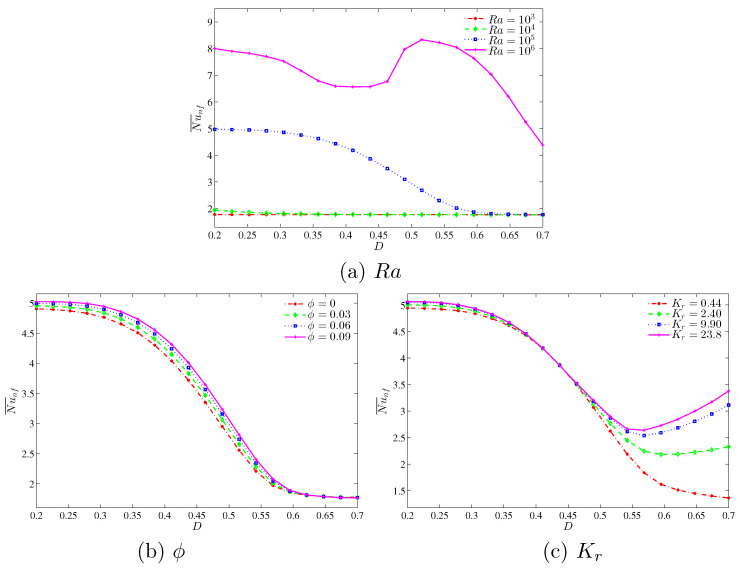
Variations of average Nusselt number with *D* for different (**a**) Ra; (**b**) ϕ and (**c**) $K_{r}$.

Figure 12 demonstrates the effect of various Rayleigh numbers, volume of nanoparticles and thermal conductivity ratios on the average Nusselt number with different inner solid lengths. We observed from Figure 12a that by increasing the length of the inner solid square, it causes the reduction in average Nusselt number at high Rayleigh numbers. This is due to the higher resistance that occurred with the increase in the inner solid length. On the other hand, at lower Rayleigh numbers, we clearly observed that the average Nusselt number is less affected with an increase in inner solid length. Figure 12b depicts the effect of various nanoparticles volume fraction on the average Nusselt number with different inner solid length. As the length of the inner solid square increases, the overall heat transfer consequently decreases for all nanoparticles volume fractions. Furthermore, we clearly observed that due to the higher thermal conductivity of nanoparticles, the convective heat transfer increases with an increase in the nanoparticles volume fraction. Figure 12c illustrates the effect of the various thermal conductivity ratio on the average Nusselt number with different inner solid length. By increasing the length of the inner cavity up to the low value (D≤0.55), the convective heat transfer decreases for all thermal conductivity ratios affected by the resistance of the solid inner cavity, which reduces the convective heat transfer. However, we can observe the increment of the average Nusselt number with an increase in *D* in [0.55,0.7], particularly for high thermal conductivity ratios.

**Figure 13:**
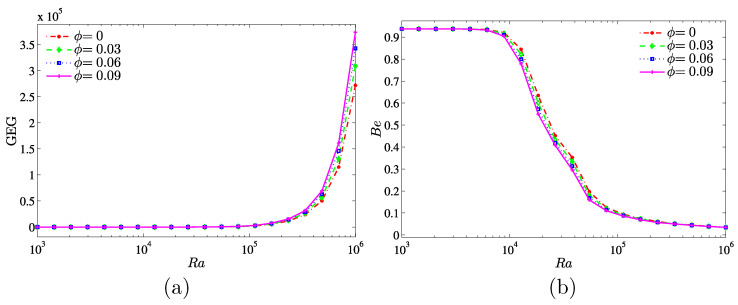
Variations of (**a**) the global entropy generation (GEG) and (**b**) Bejan number (Be) with Ra for different values of ϕ at $K_{r}=1$ and D=0.25.

Figure 13 illustrates the variation of the global entropy generation and the Bejan number with different Ra and for various nanoparticles volume fractions. In Figure 13a, the value of GEG remains constant for the low Rayleigh numbers $Ra<10^{5}$. While in the range of $Ra>10^{5}$, the GEG is increasing for all nanoparticles volume fractions especially at the higher nanoparticles volume fraction (ϕ=0.09). This is because of the enhanced inertia and viscous effects associated with the nanofluid. On the other hand, in Figure 13b, the nanoparticles inhibit the effect on the heat transfer irreversibility (HTI) for a wide range of the considered Rayleigh numbers.

**Figure 14:**
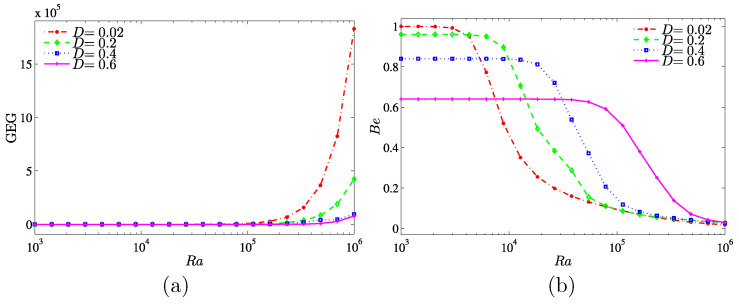
Variations of (**a**) the global entropy generation (GEG) and (**b**) Bejan number (Be) with Ra for different values of *D* at ϕ=0.04 and $K_{r}=1$.

The effects of the inner solid length are presented as a function of the Rayleigh number in Figure 14. There are no changes observed on the global entropy generation at the lower Rayleigh number for all inner solid lengths. However, at $Ra\geq 10^{5}$, the global entropy generation increases for all inner solid lengths, especially at the lower length (D=0.02). On the other hand, the Bejan number decreases with the increase of the inner solid length. The heat transfer irreversibility is dominant (Be>0.5) at lower Rayleigh number, while the nanofluid friction irreversibility (NFI) is dominance at higher Rayleigh numbers.

**Figure 15:**
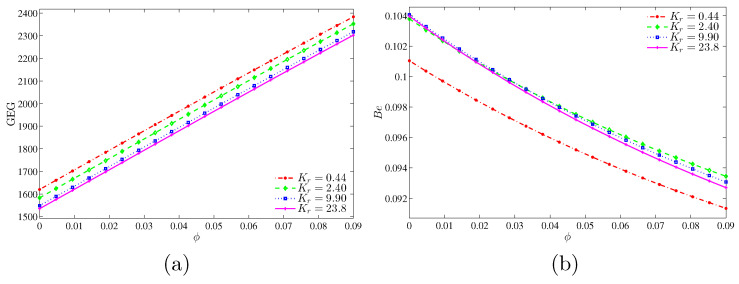
Variations of (**a**) the global entropy generation (GEG) and (**b**) Bejan number (Be) with ϕ for different values of $K_{r}$ at $Ra=10^{5}$ and D=0.25.

Figure 15 illustrates the variation of the global entropy generation (GEG) and Bejan number with nanoparticles volume fraction for different values of thermal conductivity ratio. In Figure 15a, the graph shows that the value of GEG increases with the increase of nanoparticles volume fraction for all thermal conductivity ratio. However, the GEG is higher at the low thermal conductivity ratio due to the low thermal conductivity. In Figure 15b, the graph shows that the nanofluid irreversibility is dominant for all thermal conductivity ratios. This can be imputed to the increase in the thermal conductivity, which transports more heat energy.

**Figure 16:**
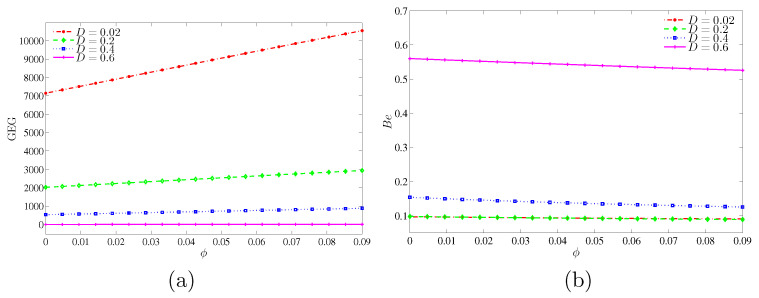
Variations of (**a**) the global entropy generation (GEG) and (**b**) Bejan number (Be) with ϕ for different values of *D* at $Ra=10^{5}$ and $K_{r}=1$.

Figure 16 depicts the variation of the global entropy generation (GEG) and Bejan number with nanoparticles volume fraction for different values of inner solid length. Only a smaller inner cavity length (D=0.02) shows an enhancement on the global entropy generation with the increase in the nanoparticles volume fraction, while at the bigger inner solid length, the global entropy generation remains the same. In Figure 16b, the Bejan number appears with a low value at the lower inner solid length while it increased with the increment of the inner solid length. It shows that the heat transfer irreversibility is dominant at the higher inner solid length whereas the nanofluid friction irreversibility (NFI) is dominant at the lower inner solid length.

**Figure 17:**
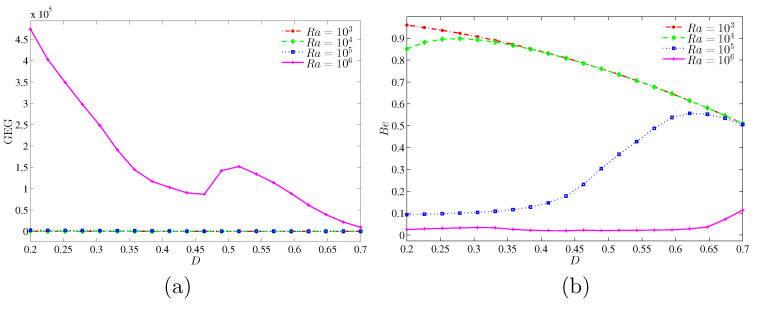
Variations of (**a**) the global entropy generation (GEG) and (**b**) Bejan number (Be) with *D* for different values of Ra at ϕ=0.04 and $K_{r}=1$.

Figure 17 shows the variation of the global entropy generation (GEG) and Bejan number with the size of the inner solid for different values of Rayleigh numbers at ϕ=0.04 and $K_{r}=1$. There has been no occurrence in enhancement on the global entropy generation at low Rayleigh numbers. However, at the higher Rayleigh number ($Ra=10^{6}$) the global entropy generation tends to enhance. The GEG is a decreasing function as the inner solid increases. In Figure 17b, we observed a high value of Bejan number with low Rayleigh numbers while the Bejan number decreases with the rise of Rayleigh number. It shows that the HTI is dominant at the higher Rayleigh number, while NFI is dominant at the smaller Rayleigh number. However, at $Ra=10^{5}$, the HTI dominant is switched to NFI as the inner solid increases.

**Figure 18:**
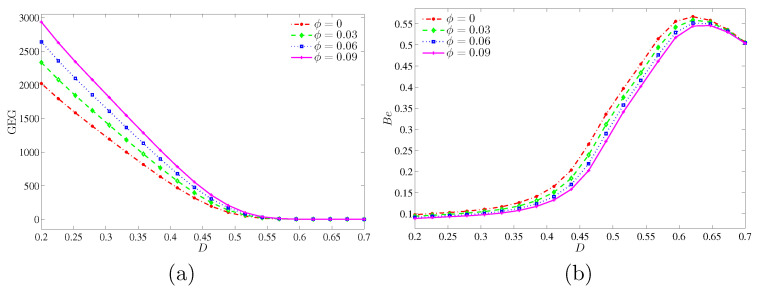
Variations of (**a**) the global entropy generation (GEG) and (**b**) Bejan number (Be) with *D* for different values of ϕ at $Ra=10^{5}$ and $K_{r}=1$.

Figure 18 presents the variation of the global entropy generation (GEG) and Bejan number with *D* for different values of ϕ at $Ra=10^{5}$ and $K_{r}=1$. This figure shows that the GEG is a decreasing function for all nanoparticles volume fractions as the inner solid increases. However, there is no changes observed on the global entropy generation for the high inner solid (D≥0.5). The global entropy generation is strong at the higher nanoparticles volume fraction due to the high thermal conductivity of nanoparticles. Meanwhile. Figure 18b shows that the Bejan number is very weak with the small inner solid and for all nanoparticles volume fractions. In addition, this figure indicates that the NFI is dominant for all nanoparticles volume fraction at the small inner solid. However, for all nanoparticles volume fraction, the system is HTI dominant at D=0.55.

**Figure 19:**
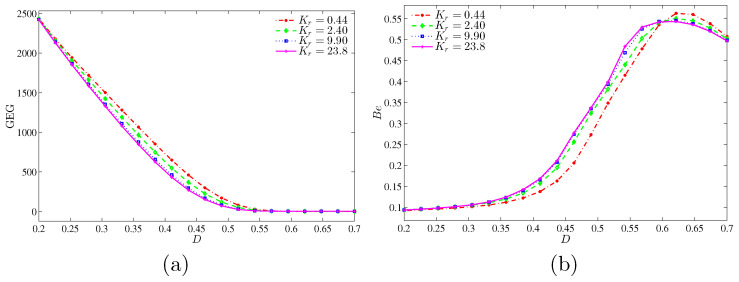
Variation of (**a**) the global entropy generation (GEG) and (**b**) Bejan number (Be) with *D* for different values of $K_{r}$ at $Ra=10^{5}$ and ϕ=0.04.

Figure 19 shows that the global entropy generation (GEG) and Bejan number with *D* for different values of thermal conductivity ratio at $Ra=10^{5}$ and ϕ=0.04. The GEG is decreasing for all thermal conductivity ratios as the inner solid increases. The GEG does not exist for the high inner solid (D≥0.55). Figure 19b shows that the NFI is dominant for all thermal conductivity ratios for a small inner solid. However, the NFI dominance is changed to HTI dominance as the inner solid reaches up to 0.55.

## 5. Conclusions

In the present numerical study, the finite difference method (FDM) is employed to analyze the entropy generation analysis and natural convection in a nanofluid square cavity with a concentric solid insert and different temperature distributions. The detailed computational results for the streamlines, isotherms and local entropy generation for different values of volume fraction of nanoparticles, Rayleigh number, thermal conductivity ratio, and inner solid length. Some important conclusions from the study are given below:

1. The inner solid insertion tends to influence the flow behavior: temperature distribution and local entropy generation affected by the resistance due to the conductive heat transfer in the solid inner square.
2. A very strong enhancement is observed on the heat transfer rate with the increasing of Rayleigh number from $10^{4}$ to $10^{5}$ as compared to the other values. This is due to a significant increment of the buoyancy forces compared to the viscous forces in this range of Rayleigh number.
3. The thermal property and the size of the inner solid square significantly influenced the rate of the heat transfer. A larger solid insert inhibits the convective heat transfer within the square cavity, as well as a low thermal conductivity of the solid insert. Furthermore, the higher thermal conductivity of the solid insert tends to increase the heat transfer rate.
4. The global entropy generation increases with the augmentation of the Rayleigh number, while a counteractive behavior was observed on average Bejan number. As Rayleigh number rises, the average Bejan number tends to reduce.
5. The global entropy generation is a decreasing function of the size of the inner solid for the case of intensive convection regime ($Ra\geq 10^{5}$), while average Bejan number is an increasing function of the size of the inner solid of the same regime.
6. A larger solid insert with a high thermal resistance inhibits the global entropy generation. However, the global entropy generation is strong at the higher nanoparticles volume fraction. Whereas a relatively bigger solid insert leads to a positive influence on the average Bejan number.
7. Some other directions in future work could include non-Newtonian fluids, three dimensional problems and non-uniform heating. Including the non-Darcy effect in the formulation of the porous medium problem is also a considerable future work.

## Figures and Tables

**Table 1:** Grid testing for $\Psi_{\min}$ and ${\bar{Nu}}_{nf}$ at different grid size for $Ra=10^{5}$, ϕ=0.04, $K_{r}=1$ and D=0.25.

Grid Size	$\Psi_{\min}$	${\bar{\mathrm{Nu}}}_{\mathrm{nf}}$
10×10	−0.76597	3.7233
20×20	−0.76613	3.7569
40×40	−0.76653	3.7802
60×60	−0.76658	3.7979
80×80	−0.76761	3.8014
100×100	−0.76878	3.8016
120×120	−0.76897	3.8018
140×140	−0.76956	3.8019
160×160	−0.76968	3.8019

**Table 2:** Thermo-physical properties of the base fluid (water) with Al${}_{2}$O${}_{3}$ nanoparticles at T=295 K [27].

Physical Properties	Fluid Phase (Water)	Al${}_{2}$O${}_{3}$
Cp(J/kgK)	4179	765
$\rho \;(\mathrm{kg}/m^{3})$	997.1	3970
$k\;({\mathrm{Wm}}^{-1}\cdot K^{-1})$	0.613	25
$\beta \times 10^{5}\;\left(1/K\right)$	21	0.85
$d_{p}\;\left(\mathrm{nm}\right)$	3.85	36

## Nomenclature

Be	Bejan number
$C_{p}$	specific heat capacity
$d_{bf}$	equivalent diameter of the a molecule of base fluid
*g*	gravitational accleration
GEG	Dimensionless global entropy generation
*k*	thermal conductivity
$K_{r}$	square wall to base fluid thermal conductivity ratio, $K_{r}=k_{w}/k_{bf}$
*L*	width and height of enclosure
*d*	width and height of inner solid square
*D*	ratio of the length of the inner solid to that of the outer hollow, D=d/L
$N_{\mu }$	irreversibility distribution ratio
$\bar{Nu}$	average Nusselt number
Pr	Prandtl number
Ra	Rayleigh number
$S_{gen}$	Entropy generation rate
$S_{GEN}$	Dimensionless entropy generation rate
$S_{\theta }$	dimensionless entropy generation due to heat transfer irreversibility
$S_{\Psi }$	dimensionless entropy generation nanofluid friction irreversibility
*T*	temperature
$T_{0}$	reference temperature (293K)
*u*, *v*	velocity components in the *x*-direction and *y*-direction
*U*, *V*	dimensionless velocity components in the *X*-direction and *Y*-direction
*x*, *y* and *X*, *Y*	space coordinatesand dimensionless space coordinates
**Greek symbols**
α	thermal diffusivity
β	thermal expansion coefficient
θ	dimensionless temperature
μ	dynamic viscosity
ν	kinematic viscosity
ρ	density
ϕ	solid volume fraction
ψandΨ	stream function and dimensionless stream function
ωandΩ	vorticity and dimensionless vorticity
**Subscripts**
*c*	cold
bf	base fluid
*h*	hot
nf	nanofluid
sp	solid nanoparticles
*w*	inner solid wall
